# Postharvest Microwave Drying of Basil (*Ocimum basilicum* L.): The Influence of Treatments on the Quality of Dried Products

**DOI:** 10.3390/foods11071029

**Published:** 2022-04-01

**Authors:** Laura De Martino, Lucia Caputo, Giuseppe Amato, Marco Iannone, Anna Angela Barba, Vincenzo De Feo

**Affiliations:** 1 Department of Pharmacy, University of Salerno, Via Giovanni Paolo II 132, 84084 Fisciano, Italy; ldemartino@unisa.it (L.D.M.); lcaputo@unisa.it (L.C.); gamato@unisa.it (G.A.); miannone@unisa.it (M.I.); defeo@unisa.it (V.D.F.); 2 EST Srl, University Spin-Off, Via Circumvallazione n.39, 83100 Avellino, Italy; 3 Institute of Food Science, CNR, Via Roma, 83100 Avellino, Italy

**Keywords:** *Ocimum basilicum* L., process sustainability, drying methods, microwave heating, essential oil

## Abstract

Edible herbs are widely used in the human diet due to their pleasant flavors and countless health benefits associated with their components having, mainly, antioxidant and anti-inflammatory therapeutic functions. Since herbs are highly perishable materials because of their high water content, to guarantee products are safe and stable over time, it is necessary that they undergo stabilization operations. The application of microwave-assisted drying, a promising technique in terms of process sustainability, for the stabilization of the aromatic herb, *Ocimum basilicum* L., was investigated. The activities were carried out by applying different operating conditions in order to evaluate the impact of the time/temperature combination on the final quality of dried basil. The latter was investigated via the chemical characterization of extracted essential oils and tissue damages. Conventional convective processes were also applied to perform comparisons between dried basil products both under production and the quality preservation points of view. Results showed that microwave heating is suitable as a drying method, as expected, due to the well-known interaction between vegetable tissue (rich in water) and the electromagnetic field; and that drying methods have a different influence on the chemical composition of the essential oils extracted from dried products, in terms of the number (ranging from 41 to 18 components in different dried samples) and percentage (until 67% in linalool and 21% in α-*trans*-bergamotene in different dried samples) of its’ constituents.

## 1. Introduction

Edible herbs are widely used in the Mediterranean Diet, considered one of the healthiest in the world, and recognized in 2010 by UNESCO as an “*intangible heritage of humanity*” [1]. The consumption of herbs contributes to increasing the quality of this diet due to the presence of secondary metabolites (terpenes, phenolics, nitrogen compounds) and the countless benefits associated with them [2]. The richness of the diet is based on the seasonality of the products used and on biodiversity. Since herbs are highly perishable materials, to obtain products safe for human health and stable over time, it is necessary that they undergo stabilization operations. The most used stabilization technique is drying, a unitary operation through which the moisture is removed from the fresh vegetal structure by a gaseous phase (air), through the simultaneous transfer of energy (heat) and matter. Drying has been applied since ancient times as the main conservation strategy for medicinal and aromatic herbs to preserve them from fermentation, mold, color and organoleptic variations due to enzymatic or non-enzymatic reactions by water activity reduction [3].

The most common herb drying techniques are based on convective heat and mass transfer. Using drying techniques under environmental conditions is applicable only in the presence of favorable climates, i.e., in sunny and breezy areas such as tropical and sub-tropical countries where, moreover, the rural economy is still present [4]. Mild environmental temperatures prevent the loss of heat-sensitive compounds, but stabilization in this way can require long waiting times, which can favor increases in metabolic and enzymatic reactions in postharvest products, and also damages caused by weeds [4,5]. Moreover, large surfaces are needed and, finally, drying treatment could not be uniform, increasing the risk of contamination. Drying in convective equipment (hot-air drying), the most widespread technique currently applied in industries, allows us to have control of the full conditions during the entire drying operation. The final quality of the plant material, in terms of taste, color and nutritional content, derives from compromises between process temperature and time.

High use of energy is required due to the intrinsic characteristics of the drying operation. To overcome the limits of quality degradation, advanced techniques are proposed. As reported in the review of Thamkaew and coworkers, 2021, [4], many examples of research with different approaches are ongoing to determine suitable solutions for the drying of heat-sensible materials such as herbs. For instance, freeze-drying allows us to obtain final products with excellent quality, but the process costs, including human resources and time, make it a suitable solution only for products with high added value, namely pharmaceutical products [6]. To overcome high costs and, under some conditions, quality degradation, the emerging technology of microwaves arises as a sustainable drying approach. Microwave heating application in the agri-food industry, for sterilization, pasteurization, tempering, pre-cooking, cooking and drying purposes, is relatively recent and not widespread. This is mainly due to the requirements of equipment and procedures often far from conventional systems (based on convective approaches) [6,7]. Nevertheless, because of the relevant advantages of microwave technology, such as rapid and selective heating and possible material quality improvements, many academic and industrial studies are currently supported and encouraged [3,8,9]. Microwaves are electromagnetic radiation with frequencies ranging from 300 to 0.3 GHz; for industrial purposes, 0.915 GHz and 2.45 GHz are the authorized and used frequencies [8,9]. Reasons for the growing interest in microwave heating consist in the mechanism high-efficiency energy transfer: it is directly delivered to materials through molecular interactions with the electromagnetic radiation through the dissipation of the electrical field into thermal energy. In this manner, it is possible to reach a high temperature in a short time inside the treated materials and, in turn, reduce manufacturing time and costs due to energy saving (i.e., microwave heating can be used as a tool for process intensification). Finally, microwave technology today, due to the easy acquisition of electrical components, is characterized by versatility and easy scalability in transferring the drying layout from the lab scale to industrial practice, allowing us to treat large quantities of materials by continuing processes [9].

In this study, the effect of microwave heating on basil was performed. The target of the investigations is the impact of heating on the global qualities and chemical composition of basil essential oil. *Ocimum basilicum* L., commonly known as sweet basil, is an annual species belonging to the Lamiaceae family, typical of the subtropical regions of Asia, Africa and South America and nowadays cultivated worldwide [3,10]. Traditionally the plant is used in medicine for the treatment of coughs, headaches, constipation, worms, kidney malfunctions and other disorders [11,12,13]. Moreover, sweet basil is, to date, one of the most popular culinary herbs used in nearly every part of the world to add a distinctive aroma to food. In general, more than 200 components are found in basil essential oil, including terpenes and terpenoids, flavonoids and aromatic compounds [10]. In Italian sweet basil, the available literature reports eugenol, methyleugenol, eucalyptol and linalool as the main constituents, which determine its particular aroma and flavor [3,14]. Basil is sold and consumed both fresh and dried but due to its very perishable nature and due to the people’s desire to cook using this plant even out of season, with organoleptic characteristics as close as possible to the fresh product, the demand for its postharvest dehydrated form is increasing on the market [15]. In this regard, the recent literature relative to the sweet basil dehydration process highlights the great potential of microwave technology (used alone or in combination with the convective methods), which, compared to the conventional techniques, allows us to: (i) reduce the drying time of basil leaves by 98.5% and 79.5% for *Ocimum basilicum* [16] and *O. gratissimum* L. [17,18], respectively; (ii) ensure high microbial quality, offering the lowest contamination by mesophilic bacteria [19]; (iii) guarantee a better aroma quality and excellent sensory data of the dehydrated product [3]; (iv) optimize the sweet basil’s drying kinetics without modifying its antioxidant properties and phenol content [11].

In order to study the preservation of quality and integrity of basil, in this article, a microwave heating emerging technology was adopted. In particular, the impact of changing the main process parameters, i.e., time–power intensity combinations, was evaluated in terms of process performance and dried product quality through the chemical characterization of the essential oil. Conventional convective processes were also applied to perform comparisons between dried basil products. Unlike literature approaches, in which microwave heating is combined with other techniques, the investigative method was maintained on the criterion of evaluating only the radiative effects on the investigated matrices. This approach was considered strategic for the rapid (and inexpensive) transposition of the studies carried out from the laboratory scale to the industrial one and more responsive to the objective of sustainable development, an objective at the heart of the current socio-economic policy promoted by the United Nations Organization as a strategy “*to achieve a better and more sustainable future for all*” [20].

## 2. Materials and Methods

### 2.1. Basil Plants

**Figure 1:**
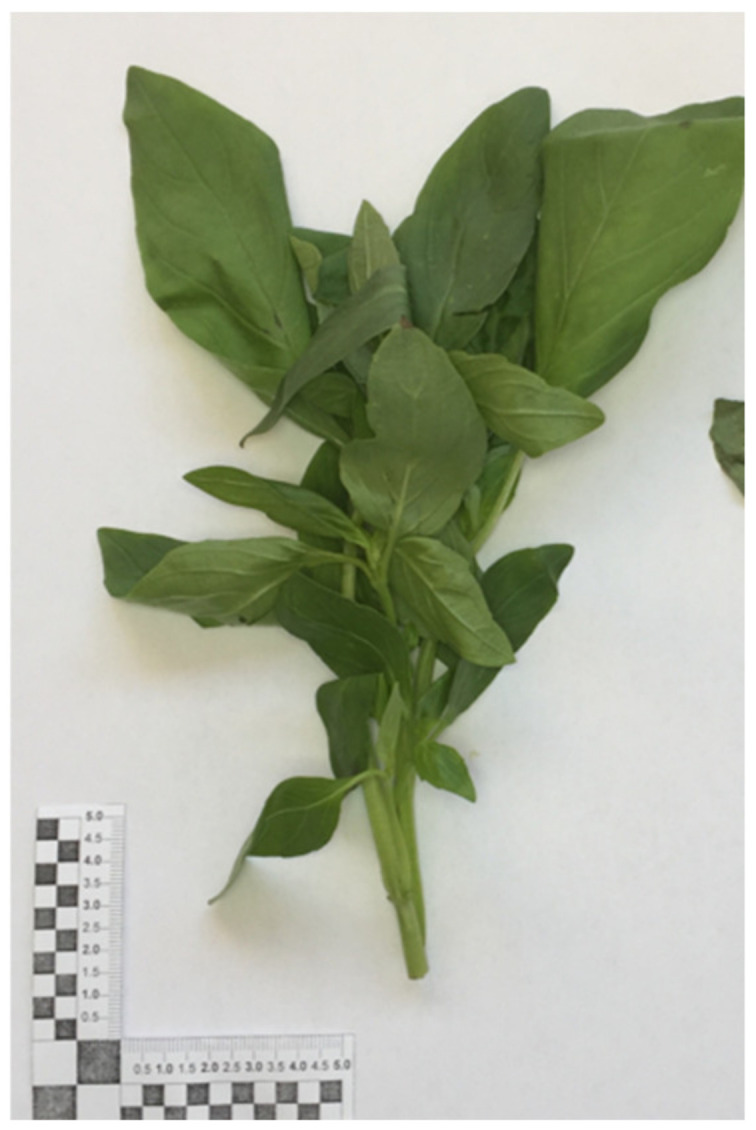
The fresh sample of *Ocimum basilicum*.

Aerial parts of *Ocimum basilicum* cv ‘Aroma 2’, were kindly supplied by the agricultural company Caselle Società Agricola (Pontecagnano, Salerno, Italy). The plants were collected in June 2020 (growth occurred at temperate clime of May–June: average temperature of 25 °C; relative humidity 60%), and a voucher specimen, identified and labeled as DF/2020/311, was deposited in the herbarium of the Medical Botany Chair of the University of Salerno. In Figure 1, a sample of the fresh product is shown.

### 2.2. Basil Samples Characterization

*Water contents*. Basil aerial parts, both fresh and dried, were subjected to moisture content (MC) measurements using the Ohaus moisture analyzer (Merck, Germany). The moisture content was defined as (water content on a wet basis):

(1) $$MC,\;\%=\frac{wet\;material\;weight-dry\;material\;weight}{wet\;material\;weight}\cdot 100$$

All the measurements were performed in triplicate, and the results are reported as average values with standard deviation SD.

*Sample visual observation*. Basil aerial parts before and after drying treatments were observed to judge and compare macroscopic differences. Leaves, inflorescences and twigs, in particular, were photographed with the aim to compare color change and shrinkage effects.

### 2.3. Drying Treatments

Basil was subjected to conventional convective and microwave heating treatments. As described in the following, operative conditions were defined after preliminary experimental runs or replacing literature indications. In Table 1, sample codes and notes on the used operative conditions are summarized.

Convective drying at aerated natural shady conditions (CD1) was performed, positioning a bed of roughly 3–4 cm of basil on a plane surface (batch weight 1 kg). It was covered with filter papers and left to dry at room temperature (25 ± 5 °C) for 6 days (after this time, moisture content was assayed). Hot-air convective drying (CD2) was carried out in an oven (ISCO series 9000, ProgiTec, Catania, Italy) always using a layer of 3–4 cm of fresh material placed on a suitable grid (batch weight 0.5 kg). The applied drying conditions were: static mode, 50 °C, 24 h [17,19]. Microwave-assisted drying was always performed using a basil bed of roughly 3–4 cm centimeters placed on netting support so as not to interact with the microwaves (batch weight 0.3 kg). The multimodal microwave cavity LBP 210/50 Microwave Oven 2300 W, InLand, Chicago, IL, USA, was managed by the True-To-Power™ system to continuously vary the power supply (operative frequency of 2450 MHz), and two internal mode stirrers were used. The surface temperature of the treated products (or under treatments) was monitored with a TASI TA601B infrared thermometer. Several pre-irradiating tests were carried out, changing the power level and radiating time and measuring the residual water content achieved (data not shown). In this way, the basil thermal behavior was observed, and the final (suitable) operative conditions were defined: 2300 W and 8 min (MWD1)—max power-; 1150 W 30 min (MWD2)—mild power-. It is important to note that the used multimode cavity allows us to irradiate without pulsed modality (i.e., with continuous electromagnetic field emission) and to work at a power level closer to industrial apparatuses than commercial devices used in many literature papers. The target of the drying activity was to achieve a residual moisture content of an average value of 10% wb [21]. After this stage, all batches of dried basil were subjected to hydrodistillation.

### 2.4. Quality Parameters

#### 2.4.1. Tissue Characterization

Drying processes can enable tissue damages which are evaluable by determining the increase in the ion (minerals or electrolytes leakages) release rate when a given amount of vegetal structure is placed in a dissolution medium with known and constant characteristics. In this study, electrolyte leakages from basil tissues were determined by conductivity measurements (by conductivity meter GLP31, Crison, L’Hospitalet de Llobregat, Barcelona, Spain) of aqueous bulk samples composed of dried (and fresh, 0.30 g) products placed in 40 mL of distilled water. Measurements were performed after 1, 2, 3, 4 h, times selected in order to observe a significant (detectable) increase in conductivity, up to a final value “of saturation” (24 h or 48 h), for which both effects of cracking and structural flaking contribute to the mineral losses in the aqueous bulk.

The change in conductivity was reported as the difference between the conductivity measured at time *t* and the initial conductivity *t_0_* (ΔC = C*t* − C*t*_0_). The results, expressed in µS cm^−1^, are reported as average values of three measurements with standard deviation (SD).

#### 2.4.2. Essential Oils

*Isolation of the Volatile Oil*. Fresh and treated basil aerial parts (with residual moisture reported in Table 2) were ground in a Waring blender and then subjected to hydrodistillation for 3 h according to the standard procedure described in the European Pharmacopoeia [22]. The essential oils were solubilized in *n*-hexane, filtered over anhydrous sodium sulphate and stored under N_2_ at + 4 °C in the dark until analyses were undertaken.

*GC-FID Analysis*. Analytical gas chromatography was carried out on a Perkin-Elmer Sigma-115 gas chromatograph equipped with FID and data handling processor. The separation was achieved using an HP-5 MS fused silica capillary column (30 m × 0.25 mm i.d., 0.25 μm film thickness). Column temperature: 40 °C, with 5 min initial hold, and then to 270 °C at 2 °C/min, 270 °C (20 min); injection mode splitless (1 μL of a 1:1000 *n*-hexane solution). The injector and detector temperatures were 250 °C and 290 °C, respectively. The analysis was also run using a fused silica HP Innowax polyethyleneglycol capillary column (50 m × 0.20 mm i.d., 0.25 μm film thickness). In both cases, helium was used as a carrier gas (1.0 mL/min).

*GC/MS Analysis*. Analyses were performed on an Agilent 6850 Ser. II apparatus, fitted with a fused silica DB-5 capillary column (30 m × 0.25 mm i.d., 0.33 μm film thickness), coupled to an Agilent Mass Selective Detector MSD 5973; ionization energy voltage 70 eV; electron multiplier voltage energy 2000 V. Mass spectra were scanned in the range 40–500 amu, scan time 5 scans/s. Gas chromatographic conditions were as reported in the previous paragraph; transfer line temperature, 295 °C.

*Identification of the Essential Oil Components*. Most constituents were identified by gas chromatography by comparison of their Kovats retention indices (Ri) (determined relative to the tR of *n*-alkanes (C10–C35)), with either those of the literature [23,24,25,26] and mass spectra on both columns with those of authentic compounds available in our laboratories by means NIST 02 and Wiley 275 libraries [27]. The components’ relative concentrations were obtained by peak area normalization. No response factors were calculated.

## 3. Results and Discussion

### 3.1. Drying Treatment Performances

The data on the moisture content of the fresh and dried basil samples are summarized in Table 2.

Basil dehydration, by lowering its moisture content, guarantees the microbial safety of the plant, preventing nonreversible chemical reactions and avoiding postharvest losses. This also extends the product’s shelf-life, allowing it to be available out of season and enabling its functional transport all over the world in reduced packaging and thus in a profitable way [19,28].

**Figure 2:**
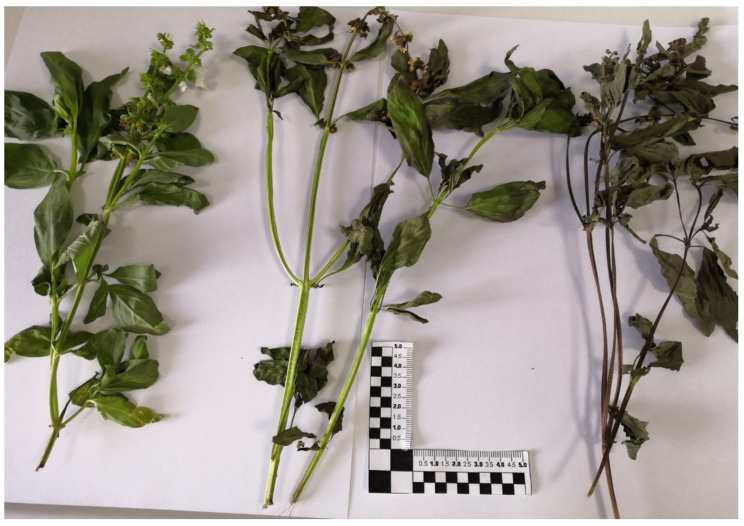
Comparison between microwave dried basil samples (center, MWD1) and convective method (right, CD2) and fresh basil (left).

Qualitative inspections of basil matrices before and after stabilization treatments were performed. The fresh basil presented a vivid color, intense smell, abundant leaves and flowers supported by bright green branches. Samples dried with the convective method in room conditions appeared withered, not excessively browned and clearly still with high moisture content. The hot air-drying process facilitated browned and fragile samples (qualitative observation was performed by rupture tendency with spatula strokes) with a high level of shrinkage; the typical aroma was attenuated. After drying assisted by microwaves, the samples had better-retained color and structure: the level of shrinkage was reduced, and the loss of color and aroma was limited [9]. Photos of the fresh and dried basil samples are reported in Figure 2. The observed differences are attributable to the different involved heat and mass transport phenomena. In microwave-assisted drying processes, samples are subjected to less thermal stress: even if they are exposed to high temperatures (MWD1:60–65 °C; MWD2:55–60 °C, hot spot values), the exposure times are considerably shorter (minutes rather than hours as at 50 °C in an oven). Reduced thermal effects allow lower chemical degradation processes (aromas and pigments losses), preserving sensorial aspects [29]. The effectiveness and drying rate are to be found in the fact that the fresh herbs exhibit a high interaction with microwaves due to their moisture content. Literature studies on the behavior of plants, including the aromatic ones, such as *Cymbopogon nardus* (L.) Rendle, *Eucalyptus* spp., *Piper aduncum* L., *Piper hispidinervum* C. DC., *Cupressus sempervirens* L. and *Cistus* spp., show how their dielectric properties, even if they are not high such as pure water medium, allow rapid heating due to the presence of a mixture of ions, non-polar long chains and large polar molecules, typical chemical constituents of branches, leaves and inflorescences, that activate dissipative energy mechanisms [30,31]. In particular, water and salt content play a fundamental role: the greater the quantity of water present (and saline content) in the plant, the better the response to the applied electric field will be and, consequently, the faster the heating and the removal of moisture. Moreover, the good electric field–plant matrix interaction also depends on external conditions such as the operating frequency, used power and macroscopic characteristics of the materials (densities, surface, thickness) [7,8].

### 3.2. Tissue Integrity Evaluation

**Figure 3:**
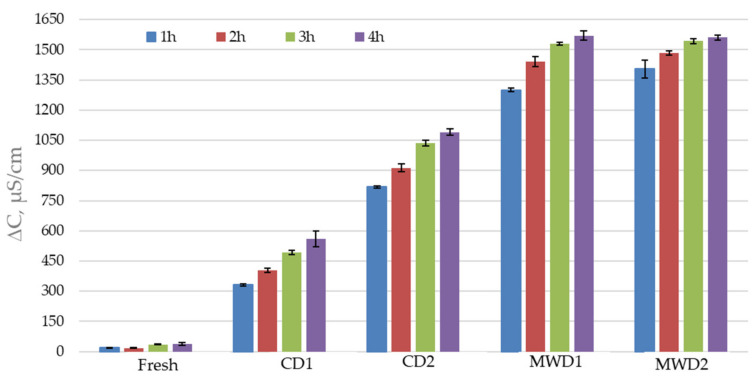
Electrolyte losses from fresh and dried basil samples (CD1—shade drying conditions; CD2—hot air-drying; MWD1—assisted microwave heating 2300 W; MWD2—assisted microwave heating 1150 W).

The high rate of mass transfer (moisture migration from herbs to the process environment) can cause tissue damage such as cracking events. To assay this, the evaluation of electrolyte loss is one method adopted as an indirect measure of cell wall integrity [32,33,34,35]. Tissue alterations of fruit and vegetables due to thermal or mechanical injuries for storage or treatment purposes (very low or high temperatures involved in freezing or in drying; washing and centrifugation) can result in an increase in the ions’ release rate from the vegetal structures in a dissolution medium [36,37]. In this work, to evaluate the effect of drying methods on tissue structure preservations of basil dried samples, ion losses in distilled water were investigated. In Figure 3, the results of electrical conductivity measurements are summarized. One-way analysis of variance ANOVA was used to compare the results referring to treated and fresh samples and treated CD and MW samples (significantly different at *p*-value < 0.05; *p* > 0.05 similar values). With respect to the fresh samples, all the dried samples’ conductivity values showed *p* < 0.05.

For fresh basil samples, the electrolyte losses in aqueous bulk were minimal due to the absence of treatment and high moisture content in the plant structure that contrasts permeation/soaking phenomena (mass transfer—water, through a different mechanism, can occur in the presence of driven force; in this case, it was absent due to the freshness of product [38]). Drying under room conditions (CD1) involves lower leakage than the other drying methods applied in this study (CD1-CD2, CD1-MWD1, CD1-MWD2: *p* < 0.05). Once again, the achieved results can be reasonably interpreted in the light of matrices with a lot of moisture present but which begin to release minerals since degenerative phenomena, due to exposure to air for 6 days, have already begun in the structures. Hot-air drying (CD2) causes greater tissue damage with respect to the convective one under room conditions due to exposure to higher temperatures (50 °C). Microwave-assisted drying treatments, both at 2300 W and 1150 W (MDW1-MDW2: *p* > 0.05), cause the most intense damage to vegetal structures, as shown by the fast and high losses of electrolytes due to the peculiar action of heat and mass transfer phenomena previously described [9,34,35]. However, this result should not necessarily be seen as a negative effect. Plant structure damage can actually facilitate extraction operation processes, and for this reason, drying appears as a suitable pretreatment process to improve process yields [9,39]. Still, to observe the impact of thermal treatments on electrolyte losses, measures of conductivity were also performed, prolonging the process times (CD1 50 °C for 48 h, MW 2300 W for 16 min). The results (not shown) confirm an increase in mineral losses. 

### 3.3. Chemical Composition of the Essential Oils

Table 3 report the yield and variation in *O. basilicum* essential oils obtained from the samples produced by the four different drying methods compared to the essential oils obtained from the fresh plant. The yields of essential oils, i.e., the ratio between the weight of the distilled plant material and the volume of the obtained Eos, is expressed as a *w*/*v*, %, change from 0.1% (fresh sample) to 1.1% (MWD2 sample). Altogether, 41 constituents were identified, accounting for 92.2–98.0% of the total oils.

Linalool (ranging from 41.7% to 65.1%) was the main constituent in all samples except for the MWD2 sample, in which α-*trans*-bergamotene resulted as the principal component with a percentage of 21%. Linalool as the main constituent was also found in several studies regarding the chemical composition of *O. basilicum* essential oils [40,41,42]. Other compounds present in lesser amounts were: 1,8 cineole (4.6–12.3%), thymol (0.2–8.4%), eugenol (0.5–12.2%), *cis*-muurola-4-(14),5-diene (0.8–11.6%), *trans*-muurola-4-(14),5-diene (1.3–8.8%) and 1-*epi*-cubenol (1.8–14.6%). These results are in accordance with our previous investigations on commercial basil essential oil [43] and the essential oil from the fresh aerial parts of *O. basilicum* cv ‘Aroma 2’ [44].

The highest number of constituents, 38, was detected in the essential oil obtained after the investigation under shady room conditions for six days (CD1), while in the essential oil obtained from assisted microwave heating/1150 W 30 min (MWD2) sample, only 18 components were found. This may reasonably be due to the high temperature reached in MWD samples compared to CD1 samples and to their high mass transfer rate (water) from the basil matrices: these effects allow for the loss of more volatile compounds. This evidence is also found in other researches on aromatic herbs [45].

As shown in Table 3, there are many statistically significant differences between the essential oils obtained by different drying methods with respect to the essential oil obtained from fresh aerial parts. In the literature, this evidence is documented and referred to both as possible hydrolytic processes and to oxidation reactions occurring during drying treatment [46]. In this study, some constituents were present in significantly greater quantities in the fresh sample than in the dried samples (highlighted with* in Table 3): δ-3 carene was present in a lesser amount in CD2 and MWD1 with respect to the fresh sample. Moreover, isobornyl-acetate and (E)-caryophyllene percentages were lower, respectively, in the CD2 and CD1 samples than in the fresh sample. Vice versa, several constituents were present in significantly lesser amounts in the fresh sample than in the dried samples (highlighted with° in Table 3): *allo*-aromadendrene, *cis*-muurola 4-(14)-5 diene, aristolochene and 1-*epi*-cubenol were present in higher percentages in all dried samples with respect to the fresh material. Thymol, γ- gurjunene and γ- muurolene were found in major quantities in the MWD1 and MWD2 samples; *trans*-muurola 4-(14)-5 diene was present in a major percentage in the CD1, MWD1 and MWD2 samples with respect to the fresh sample.

**Figure 4:**
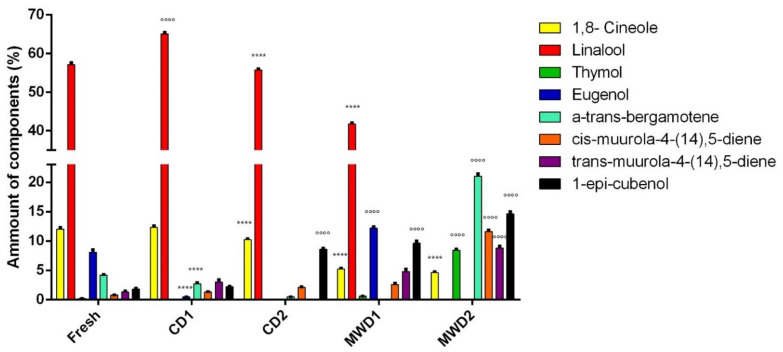
Comparative aroma profile with respect to the major components of *O. basilicum* cv ‘Aroma 2’ essential oils obtained after drying under different conditions. Statistical references: **** (component decreases) = *p* < 0.0001 vs. fresh sample according to two-way ANOVA followed by Dunnett’s multiple comparisons test, at 5% level probability; °°°° (component increases) = *p* value < 0.0001 vs. fresh sample according to two-way ANOVA followed by Dunnett’s multiple comparisons test, at 5% level probability.

Interestingly, 1,8 cineole, linalool, eugenol and α-*trans*-bergamotene showed different amounts in relation to drying methods with respect to the fresh plant (Figure 4): 1,8 cineole decreased in the CD2, MWD1 and MWD2 samples and increased in the CD1 sample. For linalool, a decrease in the CD2 and MWD1 samples and an increase for the CD1 sample were observed; a decrease of eugenol and α-*trans*-bergamotene, respectively, in the CD1 sample and both in the CD1 and CD2 samples, and an increase in the same compounds was assayed in MWD1 and MWD2, respectively. Finally, other constituents were completely absent after some treatments but were present in others. For example, myrcene was present only in the essential oil obtained with the CD1 method; β-cubebene, β-elemene and γ-himalachene were found only in the essential oil obtained by the CD2 treatment.

As reported in Table 3, sesquiterpenes hydrocarbons changed, between different treatments, from 45.9 to 68.4% of total EOs, oxygenated monoterpenes from 21.1 to 25.0%, monoterpenes hydrocarbons from 8.3 to 19.4% and oxygenated sesquiterpenes from 8.3 to 13.3%. Sesquiterpenes hydrocarbons were the main class in all of the analyzed essential oils. Monoterpenes and oxygenated sesquiterpenes were present in all samples; instead, monoterpenes hydrocarbons were absent in the MWD2 sample.

**Figure 5:**
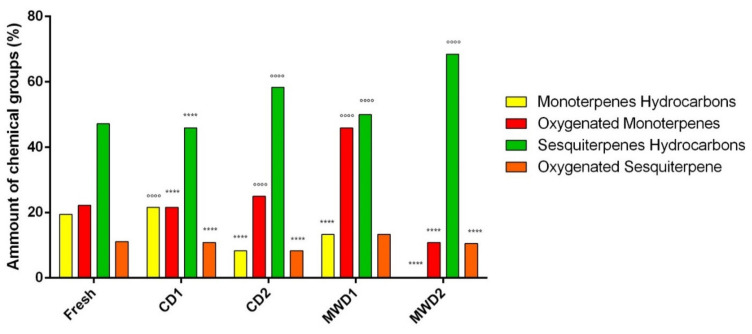
Comparison of percentages of main chemical groups (%) of fresh basil and samples dried by different methods. Statistical references: **** (component decreases) = *p* < 0.0001 vs. fresh sample according to two-way ANOVA followed by Dunnett’s multiple comparisons test, at 5% level probability; °°°° (component increases) = *p*-value < 0.0001 vs. fresh sample according to two-way ANOVA followed by Dunnett’s multiple comparisons test, at 5% level probability.

Changes in the percentages of the main chemical groups are reported in Figure 5. This variability was explained in previous literature investigations. In fact, Di Cesare and coworkers (2003), [47], using microscope examinations, showed that oil glands were broken after drying, and the retention of essential oils depends on the integrity of these glands. It was speculated that monoterpenes were driven out from the tissue during the drying process due to their lower molecular weight with respect to sesquiterpenes [48]. Moreover, as reported by Pirbalouti and coworkers (2013), [49], the drying temperature was higher in the hot air (CD2) and microwave (MWD1–MWD2) methods, so many monoterpene hydrocarbons were lost in comparison to drying at lower temperatures (CD1): in fact, monoterpenes such as α-pinene and δ-3 carene seem to be particularly temperature susceptible compounds, with greater affinity to the water fraction contained in the leaves and eliminated with water during the drying process. Moreover, in this study, an increase in sesquiterpenes in the dried basil samples was observed, as already found in the literature not only for basil [50] but also for thyme [51] and oregano [52].

Samples treated with high-temperature drying methods showed an increase in oxygenated sesquiterpenes (1-*epi*-cubenol) and sesquiterpene hydrocarbons (α-*trans*-bergamotene, *cis*-muurola-4-(14),5-diene and *trans*-muurola-4-(14),5-diene). Apparently, as also reported in the literature [49], drying led to an increased amount of water moving to the tissue surfaces, allowing the spread of components such as 1-*epi*-cubenol, α-*trans*-bergamotene, *cis*-muurola-4-(14),5-diene and *trans*-muurola-4-(14),5-diene. On the other hand, the intensity of water evaporation at the tissue surface may not have been enough to significantly increase the evacuation of those molecules, due to their high molecular mass, into the surrounding air.

## 4. Conclusions

The achieved results clearly show that the heating methodology used to obtain dried basil affects production factors and final quality, evaluated in terms of the number and percentage of constituents of extracted essential oils. Assisted microwave drying methodology undoubtedly presents the advantage of fast-drying operation, suitable for massive treatments postharvest. Furthermore, due to its characteristics of easy scalability and applicability in the continuous regime, and inexpensive installations, unlike techniques that involve combined or more complex and resources-consuming methodologies (i.e., vacuum, freeze-drying), it responds to sustainable process requirements promoted in the current socio-economic policy frame for responsible production approaches.

As an impact of assisted microwave drying on the basil matrix, there are two main evidences observed in this work. The first one is related to the yield of essential oil extraction that results in more performance using a microwave protocol (in particular MWD2); this is a relevant point considering that the production of essential oils is incredibly wasteful in terms of resources. The second point reflects the essential oil features induced by the involved heat transport phenomena. As discussed, the experimental results revealed that the applied drying method has an impact on quantitative and qualitative essential oil features: microwave protocols, in some cases, do not preserve components present in fresh or convective dried products (i.e., linalool is the main compound in all essential oil samples except in MWD2 sample where it is absent); in other cases microwave irradiating allows a higher concentration of components present in fewer amounts in fresh or diversely dried samples (i.e., in MWD2 sample: terpinolene, thymol α-*trans*-bergamotene, *cis*-muurola-4-(14),5-diene, 1-*epi*-cubenol in). The strength point of this quali-quantitative variability is related to the possibility to modify the features of the essential oil, inducing peculiar characteristics required for food purposes (flavour enhancers), cosmetic products (creams and soaps) and health uses (phytotherapeutic formulations).

## Figures and Tables

**Table 1:** Sample codes and notes on selected operative conditions of applied drying methods.

Samples Code ^1^	Drying Method/Operative Parameters
CD1	Shade drying/shady room conditions for 6 days
CD2	Hot–air drying/static oven at 50 °C for 24 h
MWD1	Assisted microwave heating/2300 W for 8 min
MWD2	Assisted microwave heating/1150 W for 30 min

^1^ Untreated product is indicated as fresh or raw material.

**Table 2:** Samples code and note on selected operative conditions of applied drying methods.

Samples Code	Residual Moisture Content % Wet Basis	Treatment Times
Fresh	85.09 ± 2.64	--
CD1	55.46 ± 8.27	6 days
CD2	10.52 ± 1.80	24 h
MWD1	10.53 ± 1.72	8 min
MWD2	10.48 ± 3.71	30 min

**Table 3:** Effects of different drying methods on the essential oil composition of *Ocimum basilicum* cv ‘Aroma 2’. Results are expressed as mean area percentage ± standard deviation (SD) of three independent determinations (*n* = 3) and statistically analyzed by two-way ANOVA followed by Dunnett’s multiple comparisons test, at 5% level probability.

*n*	Compound Name	%					KI ^a^	KI ^b^	Identif.
		Fresh	CD1	CD2	MWD1	MWD2			
1	2-Methyl butanal	0.2 ± 0.01	0.1 ± 0.01	0.5 ± 0.02 °	0.3 ± 0.01	-	745	920	1,2
2	α-Pinene	0.3 ± 0.02	0.2 ± 0.01	0.1 ± 0.01	-	-	861	1036	1,2,3
3	β- Pinene	t	t	-	-	-	873	1110	1,2,3
4	δ-3-Carene	1.1 ± 0.05	1.0 ± 0.1	0.8 ± 0.02 *	0.1 ± 0.01 ****	-	897	1153	1,2,3
5	Myrcene	-	0.1 ± 0.03	-	-	-	917	1173	1,2,3
6	1,8- Cineole	12.0 ± 0.3	12.3 ± 0.3 °	10.2 ± 0.2 ****	5.2 ± 0.19 ****	4.6 ± 0.2 ****	949	1213	1,2,3
7	β- Ocimene <Z>	0.4 ± 0.01	1.7 ± 0.1 °°°°	-	0.1 ± 0.01 *	-	970	1246	1,2,3
8	α-Terpinene	t	t	-	-	-	1001	1166	1,2,3
9	Linalool	57.1 ± 0.5	65.1 ± 0.4 °°°°	55.7 ± 0.3 ****	41.7 ± 0.35 ****	-	1020	1553	1,2,3
10	Terpinolene	3.2 ± 0.1	1.4 ± 0.1 ****	2.5 ± 0.09 ****	1.2 ± 0.1 ****	6.8 ± 0.4 °°°°	1021	1291	1,2,3
11	Isoborneol	0.2 ± 0.01	0.3 ± 0.02	0.2 ± 0.01	0.2 ± 0.01	-	1052	1633	1,2,3
12	*cis*-Dihydrocarvone	0.2 ± 0.01	0.2 ± 0.01	0.2 ± 0.01	0.3 ± 0.02	-	1075	-	1,2,3
13	*trans*-Pulegol	t	t	-	-	-	1088	1614	1,2
14	*cis*-Sabinene hydrate	0.2 ± 0.02	0.3 ± 0.02	-	t	-	1096	1470	1,2
15	Isobornyl acetate	0.3 ± 0.03	0.4 ± 0.03	0.6 ± 0.02 *	0.3 ± 0.02	0.1 ± 0.01	1185	1582	1,2
16	Thymol	0.2 ± 0.01	t	-	0.6 ± 0.03 °°°	8.4 ± 2.1 °°°°	1218	2172	1,2,3
18	δ-Elemene	0.4 ± 0.04	0.6 ± 0.04	0.8 ± 0.04 °°°	1.1 ± 0.1 °°°°	-	1228	1460	1,2,3
19	α-Ylangene	t	t	-	t	-	1241	1491	1,2
20	β-Cubebene	-	-	0.4 ± 0.01 °°°°	-	-	1264	1445	1,2
21	Eugenol	8.1 ± 0.4	0.5 ± 0.01 ****	-	12.2 ± 0.2 °°°°	-	1256	2186	1,2,3
22	Z-Isoeugenol acetate	0.8 ± 0.01	t	-	0.9 ± 0.04	-	1275	2395	1,2
23	β- Elemene	-	-	5.0 ± 0.1 °°°°	-	-	1281	1598	1,2
24	1-Ethenyl-1-methyl-2,4-bis(1-methylethenyl)-cyclohexane	2.4 ± 0.09	2.8 ± 0.1 °°°	-	6.0 ± 0.2 °°°°	6.5 ± 0.21 °°°°	1282	1593	1,2
25	(E)-Caryophyllene	0.4 ± 0.03	0.1 ± 0.01 *	-	0.6 ± 0.02	-	1285	1575	1,2,3
26	β-Ylangene	1.1 ± 0.2	1.4 ± 0.1 °	2.5 ± 0.11 °°°°	3.3 ± 0.16 °°°°	3.7 ± 0.3 °°°°	1299	1589	1,2,3
27	β-Copaene	0.6 ± 0.03	0.7 ± 0.09	1.1 ± 0.5 °°°°	1.6 ± 0.08 °°°°	0.4 ± 0.06	1312	1628	1,2
28	α-*trans*-Bergamotene	4.2 ± 0.1	2.7 ± 0.2 ****	0.5 ± 0.01 ****	-	21.0 ± 0.43 °°°°	1320	1573	1,2
29	Aromadendrene	0.3 ± 0.05	0.4 ± 0.02	-	1.0 ± 0.1 °°°°	0.7 ± 0.03 °°°	1325	1628	1,2,3
30	α-Humulene	0.5 ± 0.06	0.8 ± 0.07 °	1.3 ± 0.1 °°°°	1.6 ± 0.14 °°°°	1.5 ± 0.1 °°°°	1333	1671	1,2,3
31	*allo*-Aromadendrene	0.4 ± 0.03	0.8 ± 0.06 °°°	0.9 ± 0.03 °°°°	1.2 ± 0.11 °°°°	1.3 ± 0.09 °°°°	1343	1638	1,2,3
32	*cis*-Muurola-4-(14),5-diene	0.8 ± 0.04	1.3 ± 0.1 °°°°	2.1 ± 0.09 °°°°	2.6 ± 0.19 °°°°	11.6 ± 0,25 °°°°	1361	1675	1,2
33	γ-Gurjunene	0.3 ± 0.02	0.4 ± 0.03	0.5 ± 0.01	0.8 ± 0.04 °°°°	1.2 ± 0.1 °°°°	1367	1687	1,2
34	γ- Muurolene	0.3 ± 0.02	0.3 ± 0.02	0.4 ± 0.03	0.9 ± 0.04 °°°°	1.6 ± 0.13 °°°°	1376	1684	1,2
35	Aristolochene	0.6 ± 0.05	1.2 ± 0.1 °°°°	1.7 ± 0.08 °°°°	2.4 ± 0.4 °°°°	2.8 ± 0.21 °°°°	1387	-	1,2
36	γ-Himalachene	t	-	4.9 ± 0.15 °°°°	-	-	1391	-	1,2
37	*trans*-Muurola-4-(14),5-diene	1.3 ± 0.2	3.0 ± 0.4 °°°°	-	4.8 ± 0.4 °°°°	8.8 ± 0.3 °°°°	1395	1711	1,2
38	δ-Cadinene	0.2 ± 0.01	0.3 ± 0.01	-	0.6 ± 0.02 °°°°	-	1402	1751	1,2
39	*cis*-β-Elemenone	t	0.1 ± 0.01	-	0.1 ± 0.01	-	1484	2091	1,2
40	1,10-di-*epi*-Cubenol	0.1 ± 0.03	0.1 ± 0.02	0.3 ± 0.06	0.5 ± 0.01 °°°	0.7 ± 0.02 °°°°	1490	2054	1,2
41	1-*epi*-Cubenol	1.8 ± 0.2	2.2 ± 0.09 °°°°	8.6 ± 0.2 °°°°	9.6 ± 0.4 °°°°	14.6 ± 0.4 °°°°	1509	2025	1,2
	*Total*	99.0	99.2	98.0	98.1	98.0			
	Monoterpenes hydrocarbons	19.4	21.6	8.3	13.3	-			
	Oxygenated monoterpenes	22.2	21.6	25.0	23.3	21.1			
	Sesquiterpenes hydrocarbons	47.2	45.7	58.3	50.0	68.4			
	Oxygenated sesquiterpenes	11.1	10.8	8.3	13.3	10.5			
	Yield (*w*/*v*, %)	0.1	0.4	0.2	0.3	1.1			

* (component decreases) = *p* < 0.05; **** = *p* < 0.0001 vs. fresh sample; ° (component increases) = *p* value < 0.05; °°° = *p* value < 0.001; °°°° = *p* value < 0.0001 vs. fresh sample; a, b are the Kovats retention indices determined relative to a series of n-alkanes (C_10_–C_35_) on the apolar HP-5 MS and the polar HP Innowax capillary columns, respectively; 1 = comparison of the Kovats retention indices with published data; 2 = comparison of mass spectra with those listed in the NIST 02 and Wiley 275 libraries and with published data; 3 = coinjection with authentic compounds; t = trace (<0.1%).

## Data Availability

The datasets generated for this study are available on request to the corresponding author.
